# Multidisciplinary surgical management of severe posterior compartment endometriosis

**DOI:** 10.1007/s00464-024-10969-7

**Published:** 2024-06-19

**Authors:** Carolin Mueller, Miguel Luna Russo, Lukas Schabl, Hermann Kessler

**Affiliations:** 1 Outcomes Research Consortium, Department of Anesthesiology, Cleveland Clinic, Cleveland, OH USA; 2 Department of Gynecology, Obstetrics, and Reproductive Medicine, Saarland University Medical Center, Homburg, Saar Germany; 3 Division of Minimally Invasive Gynecologic Surgery, Women’s Health Institute, Cleveland Clinic, Cleveland, OH USA; 4 Department of Colorectal Surgery, Digestive Disease and Surgery Institute, Cleveland Clinic, Cleveland, OH USA

**Keywords:** Endometriosis, Laparoscopy, Interdisciplinary surgery

## Abstract

**Background:**

Endometriosis is a chronic, inflammatory, and hormone-dependent disease that affects approximately 10% of women in reproductive age. Endometriosis is categorized into different types, as superficial, deep, and ovarian endometriosis. When deep endometriosis occurs, the sigmoid and rectum are often affected (Becker et al. in Hum Reprod Open, 2022, https://doi.org/10.1093/hropen/hoac009). In the following article, we aim to demonstrate stepwise surgery for stage IV endometriosis involving the anterior rectosigmoid.

**Methods:**

We present the case of a 26-year-old obese (BMI 35.87) woman with severe posterior pelvic compartment endometriosis, persistent abdominal pain, and constipation. On preoperative MRI of the pelvis, a 13 cm conglomerate incorporating both ovaries (kissing ovaries), uterine serosa, and the anterior rectosigmoid was observed (Fig. 1). Accordingly, interdisciplinary laparoscopic surgery with a gynecologist and colorectal surgeon was planned.

**Results:**

The total laparoscopic approach is demonstrated step by step in the video.

**Conclusions:**

Deep endometriosis is a rare condition. When involvement of other organs (e.g., the bowel) is suspected, preoperative endometriosis-specific imaging should be performed for optimal surgical planning. Experienced endometriosis multidisciplinary surgical teams can provide specialized and high-quality care for patients suffering from this debilitating disease (Luna Russo et al. in Minerva Ginecol, 2020, https://doi.org/10.23736/S0026-4784.20.04544-X).

**Supplementary Information:**

The online version contains supplementary material available at 10.1007/s00464-024-10969-7.

## Problem

Endometriosis is a chronic benign disease. According to the WHO, up to 10% of women of childbearing age (190 million women worldwide) have endometriosis [3]. In a cross-sectional survey of American women, 6.1% of women of childbearing age were diagnosed with endometriosis [4]. However, 6 of 10 patients with endometriosis are undiagnosed [5]. Endometriosis is the presence of endometrial-like tissue outside the uterus and can occur as superficial, deep, or ovarian endometriosis [1, 2]. Deep endometriosis occurs in up to 10% of endometriosis patients [6]. 95% of deep infiltrating endometriosis affects the rectum or sigmoid, whereas other areas (e.g., appendix, ileum, bladder, and ureter) are less frequent [6]. The clinical picture of endometriosis varies, ranging from no symptoms to cyclic menstrual pain, acyclic pain, chronic pelvic pain, and infertility [1]. Moreover, the severity of symptoms in endometriosis does not necessarily correspond to the extent of the condition. Patients with superficial endometriosis may experience intense chronic pain, while those with deep endometriosis might be asymptomatic, and the reverse can also be true [1].

**Fig. 1:**
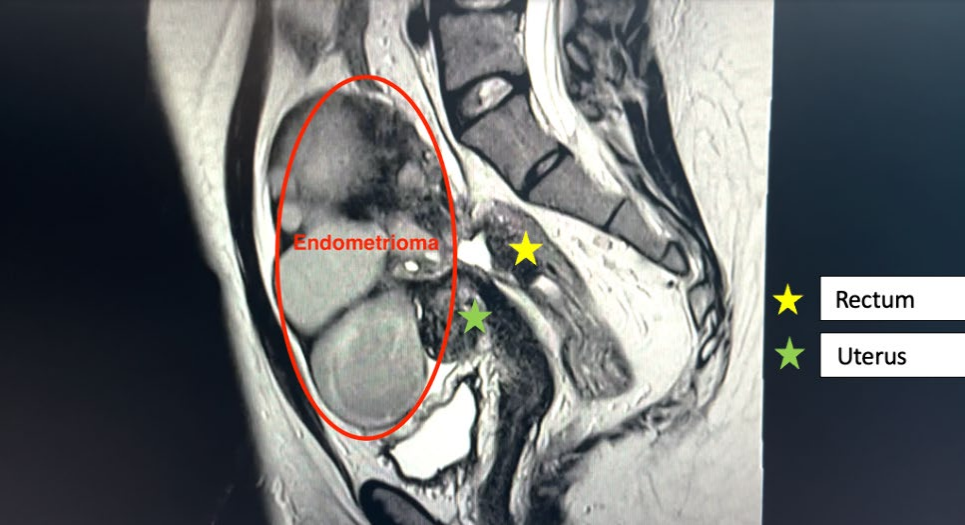
The preoperative MRI showed a 13-cm endometriosis conglomerate incorporating both ovaries, the outer uterine wall, and the anterior rectosigmoid

Herein, we present the case of a 26-year-old, obese (BMI 35.87) woman with stage IV endometriosis. She had persistent pelvic pain and significant constipation for two years. She had dysmenorrhea but no dyschezia, dysuria, or dyspareunia. Cyclic pseudobstructive symptoms were present. She had a history of migraine (without aura), gastrointestinal reflux, and a vaginal infection with chlamydia and trichomonas two years ago. Physical examination revealed that the abdomen was soft and nondistended. However, a palpable mass was observed in the right lower quadrant. Transvaginal ultrasonography revealed multicystic density of approximately 15 cm. As incorporation of other organs (e.g., bowel) could not be proven with ultrasound, MRI was performed to better assess the full extent of the lesion. MRI showed a conglomerate of 13 cm incorporating both ovaries, multiple endometriomas, and extensive deep endometriosis, including the outer uterine wall. Furthermore, two endometriotic lesions in the bowel (one in the anterior rectosigmoid and one in the sigmoid) were observed with suspected partial bowel obstruction (Fig. 1). Prior to surgery, the patient underwent flexible sigmoidoscopy. Preparation was good and the scope was inserted to a level of 30 cm above the anal verge. Granulation tissue and extrinsic compression were observed at 20 cm. The remainder of the sigmoid, rectum, and anal canal were entirely normal. The patient received norethindrone (Aygestin) 5 mg daily, for a total of 3 months prior to surgery. However, this did not improve her symptoms considerably.

## Our solution

The patient desired surgical management for endometriosis-associated pain and symptoms. Patient was thoroughly counseled regarding fertility preservation options, risk of the procedure, and alternatives to surgery. Laparoscopic endometriosis excision was planned by a multidisciplinary surgical team consisting of gynecologic surgery and colorectal surgery.

**Fig. 2:**
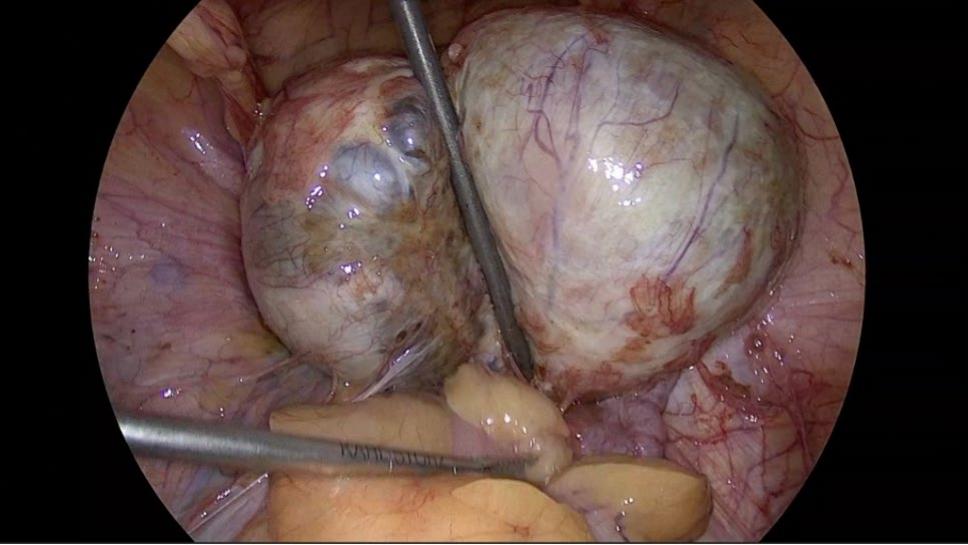
Intraoperative view at the beginning of surgery

**Fig. 3:**
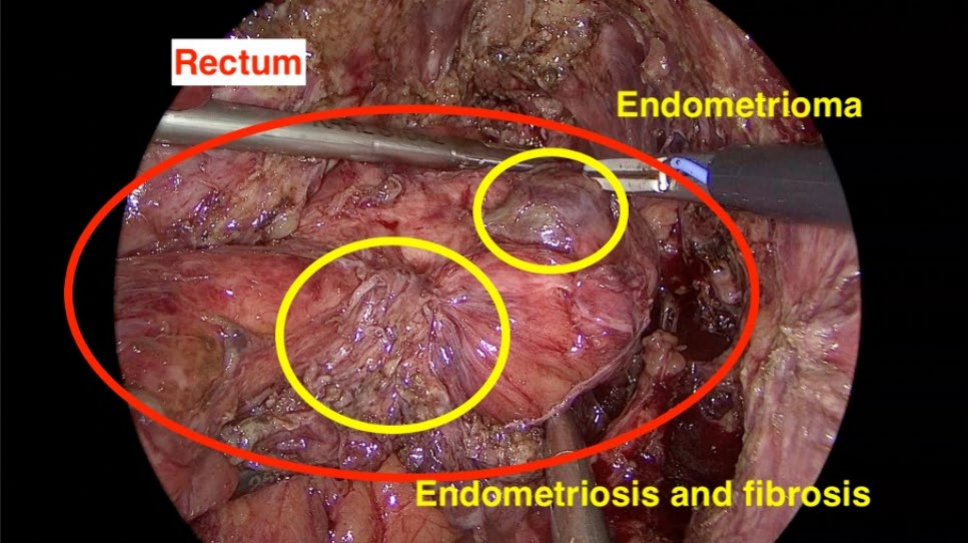
Bowel lesions

When entering the surgery at diagnostic laparoscopy, large endometrioma incorporating both ovaries and the rectum were found (Figs. 2 and 3). Given the sizeable (> 3 cm) endometrioma found on the rectosigmoid, along with another visible lesion further proximal, a segmental resection of rectum and sigmoid colon was carried out to minimize the significant risk of recurrence. Our video demonstrates the surgical approach used for stage IV endometriosis in our patient.

1. Cyst drainage (ovaries)
2. Ureterolysis
3. Separation of ovaries, preparation of uterine serosa, and rectocervical space
4. Ovarian cystectomy
5. Mobilization of rectosigmoid
6. Preparation of the distal transection point (rectum)
7. Preparation of the proximal transection point
8. Pfannenstiel mini-laparotomy incision, implanting of an Alexis device, and extraction of rectum and sigmoid colon
9. Mobilization and transection of the proximal transection point (colon)
10. End-to-end colorectal anastomosis
11. Sigmoidoscopy and air leak test

**Fig. 4:**
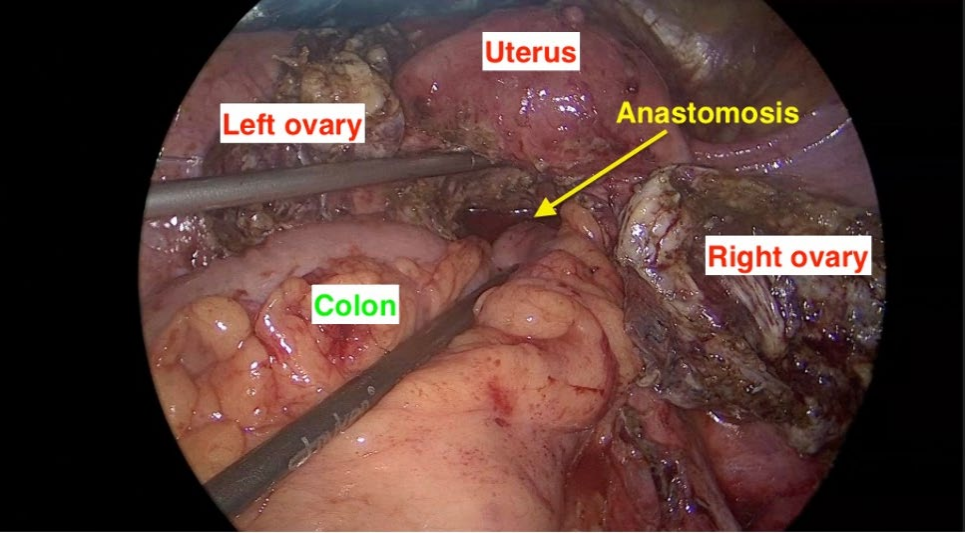
Intraoperative view at the end of surgery

At the end of the surgery, no bleeding and an intact end-to-end colorectal anastomosis were observed (Fig. 4). The patient was discharged without any complications. One month after the surgery, the patient had no abdominal pain, the wound healed well, and bowel movements were within normal limits. The patient resumed norethindrone (Aygestin) 5mg daily after surgery.

In conclusion, deep infiltrating endometriosis is a rare condition. When involvement of other organs (e.g., the bowel) is suspected, multidisciplinary surgery should be planned to avoid multiple procedures and complications.

## Summary

When deep endometriosis is suspected, advanced endometriosis specific imaging and interdisciplinary surgery should be the standard for those requiring surgical management.

### Supplementary Information

Below is the link to the electronic supplementary material.

Total laparoscopic approach in stage IV endometriosis with a step-by-step explanation. 1. Cyst drainage (ovaries), 2. Ureterolysis, 3. Separation of ovaries, preparation of uterine serosa, and rectocervical space, 4. Ovarian cystectomy, 5. Mobilization of rectosigmoid, 6. Preparation of the distal transection point (rectum), 7. Preparation of the proximal transection point, 8. Pfannenstiel mini-laparotomy incision, implanting of an Alexis device and extraction of rectum and sigmoid colon, 9. Mobilization and transection of the proximal transection point (colon), 10. End-to-end colorectal anastomosis, 11. Sigmoidoscopy and air leak test. Supplementary file1 (MP4 248260 kb)

## Data Availability

Not applicable.
